# Spontaneous testicular tumor regression: case report and historical review

**DOI:** 10.3332/ecancer.2018.888

**Published:** 2018-12-18

**Authors:** Juan C Astigueta, Milagros A Abad-Licham, Folker M Agreda, Benjamin A Leiva, Jorge L De la Cruz

**Affiliations:** 1 Oncological Urology Service, Regional Neoplastic Disease Institute, Trujillo 13007, Peru; 2 School of Medicine, Antenor Orrego Private University, Trujillo 13007, Peru; 3 Oncological Pathology Service, Regional Neoplastic Disease Institute, Trujillo 13007, Peru; 4 Centre of Excellence in Pathological Oncology, Trujillo 13007, Peru; 5 Department of Oncological Surgery, Virgen de la Puerta Hospital, Essalud, Trujillo 13007, Peru; 6 Oncological Medicine Service, Regional Hospital of Lambayeque, Chiclayo 882, Peru

**Keywords:** testicle, spontaneous regression, burned out, germ-cell tumour

## Abstract

Spontaneous regression of a primary testicular germ-cell tumour (GCT), over time known as ‘Burned out’, ‘Shrinking Seminoma’, ‘pT0’, ‘Burnout’ or ‘Spontaneous Regression’, is an uncommon, generally metastatic phenomenon, which may present elevated tumour markers and a suspicious testicular ultrasound image. The histological study of the testicle demonstrated morphological changes of complete or partial tumour regression and found fibrous scarring and other characteristic changes of this phenomenon, which in some cases include vestiges of GCT.

There are few publications on testicular GCT tumour regression and those that exist present limited data on the biology of the disease and its etiopathogenesis. This entity was recently recognised in the latest edition of the World Health Organization’s (WHO) Classification of Tumours.

We present our clinical, imaging, laboratory, cytohistological and management experience, as well as a historical review of the literature.

## Introduction

Spontaneous tumour regression has been reported in various neoplasias [1, 2]. In testicular cancer, it is defined as a germ-cell tumour (GCT) that has completely or partially regressed, without any intervention, leaving a scar in the parenchyma with or without vestiges of GCT [3]. The etiopathogenesis of the regression is not defined and it is thought that less than 5% of all testicular GCTs undergo spontaneous regression [4]. It usually presents as metastatic disease and is manifested by symptoms secondary to it. It may have high tumour markers, depending on the histological lineage. Historically, many cases have been classified as primary extragonadal GCTs (EGCTs) but most subsequent studies found evidence of regression of a primary testicular [5–24].

We present our experience and carry out a historical review of the literature.

## Materials and methods

### Sample

Clinical records of patients from the Urology Service of the Regional Institute of Neoplastic Diseases North, in Trujillo, Peru, were reviewed, from January 2010 to June 2018. We identified the cases with a diagnosis of testicular GCT regression and proceeded to collect the data in a digital card developed for this purpose.

### Epidemiological and clinical study

We obtained data such as age, pathological history, time of illness, signs, symptoms and information from the physical examinations.

### Imaging study and clinical laboratory

Imaging information obtained was confirmed by evaluating existing material in the Radiology services’ records (ultrasound, x-rays, CT scans and others). Tumour marker data such as alpha fetoprotein (AFP), human chorionic gonadotrophin (HCG) and lactic dehydrogenase (LDH) were collected and correlated with the histological findings.

### Management, evolution and current status of the disease

We collected data regarding the type of treatment (surgery, chemotherapy, radiotherapy and other), as far as primary, metastasis and/or recurrence.

### Cytological and anatomopathological material

- Of the testicle.
- Of the metastasis.

We reviewed the cytological and histological material, classifying it according to the World Health Organization’s (WHO’s) 2016 classification of testicular tumours and paratesticular tissue. This edition recognises GCT regression as an entity [4].

### Staging

We used the 8th Edition American Joint Committee on Cancer (AJCC) staging system based on the study of tumour (T), lymph node involvement (N), the presence of metastasis (M) and serum tumour marker (S) [25].

### Current state of the disease

Upon reviewing the medical history, we obtained the dates of the last check-up and the state of the disease. In the cases with no recent data, we located patients.

### Review of existing literature

Bibliographic searches were conducted on Scopus, Medline, EBSCO and BVS from 2000 to the present. The data obtained were analysed, compared and discussed.

## Results

### Sample

In the review of the medical records, five [5] cases were identified with a diagnosis of primary testicular GCT regression, all metastatic with complete regression.

### Case Report

#### Case 1

A 54-year-old patient without any significant history, with a 6-month disease time, characterised by weight loss, abdominal tumour and lumbar and abdominal pain. Upon physical examination, no peripheral adenopathies were found, a hard fixed mass was felt in the abdomen located in the mesogastrium and testicles with no notable particularities. On the CT scan of the chest, abdomen and pelvis (CT-CAP), a retroperitoneal 17 cm × 10 cm × 8 cm tumour was observed. It encompassed the abdominal aorta, collapsed the vena cava and projected to the iliac arteries; it was initially classified as *lymphoma*. A core percutaneous biopsy was performed and had inconclusive pathological results. As part of imaging studies, a scrotal ultrasound was requested and a 23 mm × 26 mm hypoechogenic nodule was found in the right testicle. The AFP and HCG tumour markers were found in normal parameters and the LDH in 2480UI. Radical orchiectomy was performed and fibrous scarring associated with histological changes of regression was found. He underwent exploratory laparotomy and subtotal resection of the retroperitoneal tumour with a cytological, histological and immunohistochemical study consistent with a seminoma-type GCT. He received chemotherapy (chemo) with complete tumour remission and tumour markers within normal values; during treatment, he developed deep vein thrombosis (DVT) in both lower extremities (LEs), which was managed medically. At the 60-month follow-up, he shows no evidence of disease.

#### Case 2

A 58-year-old patient with a 2-month disease time, characterised by bilateral lumbar pain, sensation of thermal rise, volume increase in LEs and weight loss. Physical examination revealed a left supraclavicular lymph node conglomerate of hard consistency, a mass in mesogastrium, hard edema in both legs and no other peripheral adenopathies. The abdomen and testicles were without any particular features. Supraclavicular, subclavian, mediastinal and retroperitoneal adenopathies were observed via CT-CAP, the latter being in a 15 cm × 13 cm × 9 cm conglomerate, which includes the great vessels, as well as thrombus in vena cava and iliac vessel. It was also initially classified as *lymphoma.* A fine-needle aspiration biopsy (FNAB) of the supraclavicular adenopathy was performed, revealing cytological features of a malignant round-cell neoplasm, likely seminoma. The scrotal ultrasound showed the right testicle with microcalcifications and an 8 mm × 7 mm hypoechogenic nodule. Tumour markers were AFP 4.5UI/l, HCG 8.02UI/L and LDH 3637UI/L. A radical orchiectomy was performed, with a histological report of the fibrous nodule and regression changes, among them germinal neoplasia *in situ*. A supraclavicular tumour biopsy was performed with histological and immunohistochemical study consistent with seminoma-type GCT. The patient received chemo with complete tumour remission at the supraclavicular and mediastinal level; the remission was partial in the retroperitoneum, therefore, a positron emission tomography was performed, reporting para-aortic tissue of 35 mm, inactive residual appearance, which diminished in subsequent tests. At the 42-month follow-up, there is no evidence of active disease.

#### Case 3

A 23-year-old patient with a history of thoracic trauma and haemoptysis underwent a thoracic tomography, in which multiple nodules were observed in both pulmonary fields. Subsequently, he developed hemiplegia on the left; in the supplemental CT, a 48-mm hypodense image in the brain was found in the right fronto-parietal region; also in the retroperitoneum, a 11cm × 7 cm × 4cm ganglion conglomerate. Physical examination revealed no peripheral adenopathies, resistance to palpation in the abdomen at the level of mesogastrium and testicles without particular features. The scrotal ultrasound revealed a left testicle with multiple microcalcifications and a 9 mm × 8 mm heterogeneous, hypoechogenic nodule. The tumour markers were AFP 0.9UI/L, HCG 19609UI/L and LDH 561UI/L. Radical orchiectomy was performed with pathology that showed the testicle with fibrous scar associated with histological regression changes. The patient underwent a pulmonary nodule FNA with cytological study compatible with choriocarcinoma-type GCT. Due to brain metastasis, he received radiotherapy (RT) and started chemo. During the treatment, he presented seizures, anaemia and febrile neutropenia; the poor clinical response was observed in imaging studies (hepatic metastasis, increased dimensions of brain metastasis with perilesional haemorrhage) and in the tumour markers (AFP 3.73UI/L, HCG 214UI/L and LDH 734UI/L). Chemo was not completed due to complications and the patient died 7 months after the initial diagnosis with the evidence of disease progression.

#### Case 4

A 36-year-old patient with no relevant history, with a 7-month disease time characterised by left lumbar pain, abdominal mass, weight loss and increase in volume of the left leg. Upon physical examination, no peripheral adenopathies were found. In the abdomen, a hard and fixed mass was felt in the mesogastrium, testicles had no particularities and an increase in volume in the left leg. On the CT-TAP, a retroperitoneal tumour of 16 cm × 9 cm × 9 cm was observed, which includes the aorta and the cava. Doppler ultrasonography reported DVT in the iliac, femoral and popliteal veins of the left leg. On the scrotal ultrasound, a 30-mm hypoechoic nodule was found in the left testicle, associated with multiple microcalcifications. The tumour markers were AFP 0.69UI, HCG 0.44UI and LDH 1240UI. Radical orchiectomy was performed with pathology that reported parenchyma with a fibrous scar and tubular hyalinization. Percutaneous biopsy of the retroperitoneal tumour with histological and immunohistochemical exam was compatible with the seminoma-type GCT. Full chemo with partial remission of the disease and tumour markers were within normal parameters. The residual tissue has progressively decreased in volume. Currently, at 20 months of follow-up, there is no evidence of active disease.

#### Case 5

A 20-year-old patient without any significant history, with a 6-month disease time, characterised by weight loss, abdominal tumour and pain. Upon physical examination, no peripheral adenopathies were found, a hard fixed mass was felt in the abdomen located in the mesogastrium, testicles had no notable particularities. On the chest, abdomen and pelvis CT, a retroperitoneal tumour was observed predominantly in the left iliac region measuring 16 cm × 15 cm × 9 cm, which includes iliac vessels and left renal agenesis. Suspecting a metastatic GCT, a scrotal ultrasound was performed and found a 5 mm × 10 mm isoechogenic nodule in the left testicle. Tumour markers were 1745UI AFP, HCG 3705UI and LDH 2948UI. Radical orchiectomy was performed and fibrous scarring associated with histological changes of regression was found. A percutaneous biopsy of the retroperitoneal tumour was performed, with a cytological and histological report of mixed GCT (embryonal carcinoma, teratoma and yolk sac tumour). During the evolution of the disease, one of the complications presented was a bowel obstruction, resolved with sigmoidectomy and block retroperitoneal lymphadenectomy. Patient was currently in chemo with partial remission.

### Epidemiological and clinical study

The cases presented between 20 and 58 years with an average age of 38 years. No significant data were found with regard to history. The average disease duration was 3.8 months (range 1–7 months), mainly characterised by abdominal and/or lumbar pain, weight loss and abdominal tumour in three cases and one supraclavicular tumour. On examination of testes, tumours were not felt on palpation in any patient. In two cases, the initial diagnosis was lymphoma Table 1.

### Imaging and laboratory study

**Figure 1.**
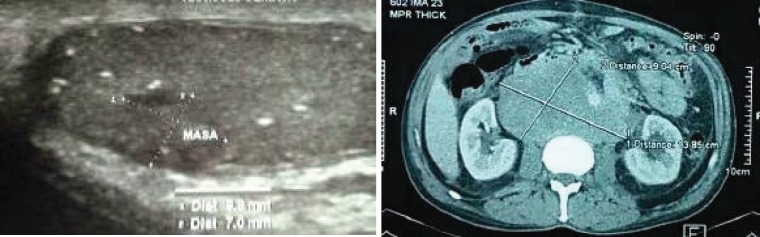
Imaging studies: (A) Ultrasound showing heterogeneous nodule and microcalcifications in the testicular parenchyma. (B) Tomography with retroperitoneal mass that encompasses large vessels.

The results of the imaging studies conducted are summarised in Table 2. All five cases were metastatic, with retroperitoneal tumours larger than 10 cm; in four cases, the tumour encompassed the aorta, cava and/or iliac Figure 1. Two had a diagnosis of DVT. As to tumour markers, in all cases, the LDH was high and, in the other two, HCG and AFP.

### Management, evolution and current status of the disease

The initial clinical suspicion in the first three cases was different from primary testicular metastatic GCT, therefore, the diagnostic work-up included aspiration and/or surgical biopsies of retroperitoneal, supraclavicular and lung masses, respectively. Laboratory studies and images were supplemented with histological findings and radical orchiectomy was performed. In the last two cases, where GCT was suggested from the beginning, the pathology of the orchiectomy was consistent with tumour regression, having found evidence of germinal neoplasia in the study of the metastasis.

With the anatomopathological diagnosis of testicular tumour regression and metastatic staging, all patients received chemo and had favourable responses corroborated through imaging studies and MT, except for the third case, who also received RT due to brain metastasis and progressed to death (Table 3).

### Anatomopathological material

The existing material was reviewed and classified according to WHO classification of testicular tumours and paratesticular tissue (4).

**Figure 2.**
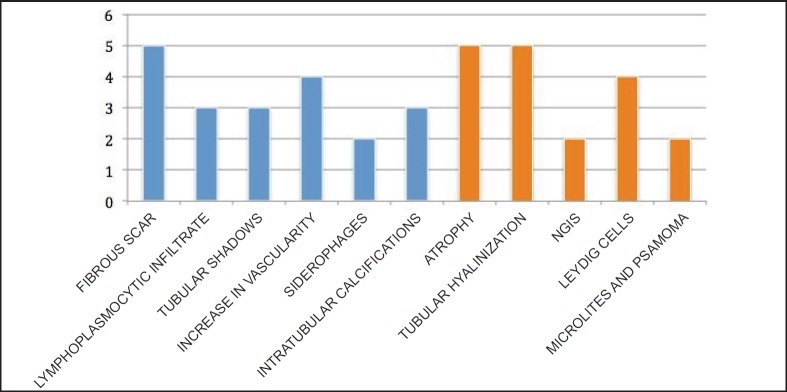
Histomorphological characteristics of testicular tumour regression: scar (blue) and paracicatricial area (orange). The case numbers are on the ‘Y’ axis.

**Figure 3.**
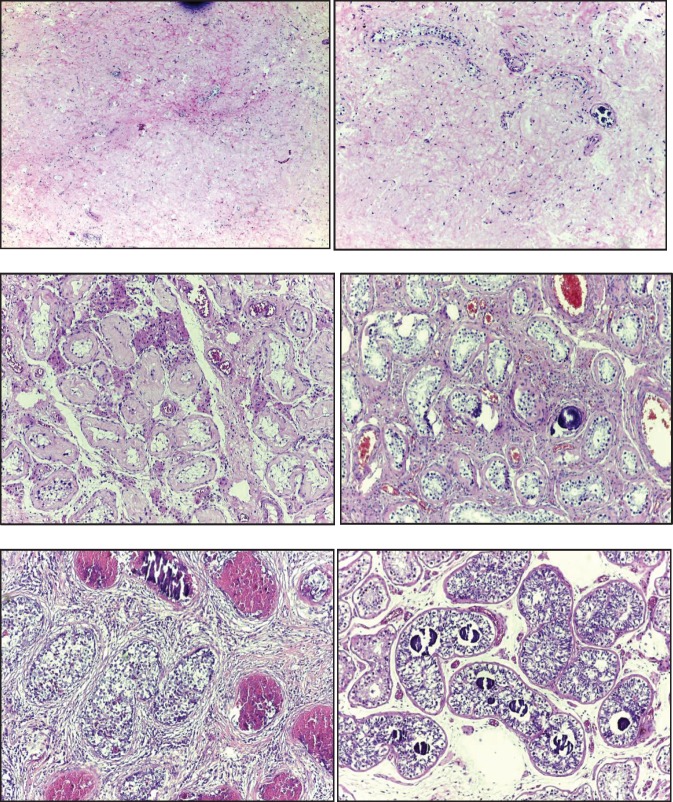
Microphotographs of the histomorphological characteristics of the testicular tumour regression: (A) Fibrous scar with increased vascularity. (B) Increase in vascularity and microcalcifications. (C) Tubular hyalinosis and presence of Leydig cells. (D) Microliths in paracicatric area. (E) NGIS-type embryonal carcinoma. (F) NGIS and intratubular calcifications.

In the ***testicle*** product of radical orchiectomy, macroscopically, all cases presented a whitish fibrous scar, located close to the to rete testis. The surrounding testicular parenchyma did not present significant alterations. For histological interpretation, we divide the testicles into two regions: the scar and the area adjacent to the scar (paracicatricial), whose characteristics are described in Figure 2 and shown in micrographs in Figure 3.

With regard to *metastasis*, four of these were evaluated initially with cytology, two with FNAB and two with intraoperative cytology. The results of these showed germ-tumour cytology, allowing identification of the types seminoma, choriocarcinoma and embryonal carcinoma. Subsequently, all cases were subjected to conventional histological and immunohistochemical study, corroborating diagnoses of germinal neoplasia; the latter presented teratoma and yolk sac tumour in addition to embryonal carcinoma. Table 4 presents the anatomopathological results in correlation with the tumour markers.

### Staging

The five cases were classified as metastatic stage pT0, with findings of testicular tumour regression.

### Literature review

Performing a search from January 2000 to June 2018, 159 cases were found in 57 articles. The cases arose between 17 and 67 years old with an average age of 35.96 years; only in nine cases (15.8%), cryptorchidism was reported as a precedent. 96.8% of the patients had metastatic disease and 71.7% had complete regression.

Likewise, the time frame of the review found that there is a discrete increased frequency of regression in the right testicle (47%) and the more common histological type is seminoma (50.8%). The publications by author’s locations were as follows: Europe 22 (38.6%), Asia 18 (31.6%) and America 17 (29.8%). The summary of the data is presented in Tables 5 and 6.

In the review conducted, there are some publications of cases reported as ‘burned out’, the same as the clinic and/or imaging studies and/or laboratory are compatible with GCT; however, they were not considered because they do not have complete data, especially histological findings of the testicle.

## Discussion

Over time, the ‘phenomenon’ of tumour regression has been described in different pathologies such as melanoma, breast cancer, lymphoma, renal carcinoma, among others [1, 2]. It is currently known that the process that keeps tumours alive does not only depend on their ability to multiply and block apoptosis, but there is also a close relationship with the immune environment in which the tumour develops, the so-called tumour microenvironment [4, 26–28].

There are not many publications on the regression of testicular GCTs; this entity has only recently been recognised in the last edition of the WHO’s book on Tumours of the Urinary System and Male Genital Organs (2016), in the chapter on testicular tumours and paratesticular tissues [4]. It is considered that the first to describe this phenomenon was Prim in 1927; he reported the case of a 51-year-old patient who died with multivisceral and retroperitoneal lymph node metastasis, with ‘chorionephiteliomatösen’ histology and no known primary. At the autopsy, he found a scar on the right testicle and posed the question of whether it may have been the primary one and presented ‘spontaneous healing’ [29]. In 1954, Rather *et al* [30] reported six new cases and reviewed the bibliography, finding 18 additional cases. In the final histological analysis, they described testicles as seven having only one scar, nine having germ-cell tumours and eight having fibrosis, tubular and cystic structures, hemosiderin deposits and calcification. The characteristics of testicular tumour regression have been defined this way for over six decades, with a high approximation for existing approaches, same as have been identified in our sequence.

In 1955, Slater *et al* [31] reported a case where a patient with a retroperitoneal mass with ‘seminoma and chorioepithelioma’ histology. He underwent bilateral orchiectomy and a small, solid nodule in the left testicle was found, which the microscope identified as scarred teratoma, confirming that they were dealing with a ‘**burned out** primary…’. This is likely *the first publication to use this terminology* to define tumour regression in germ cells.

In 1961, Azzopardi *et al* [32] published a series of 17 cases of young patients that passed away due to disseminated metastatic illnesses, eight with choriocarcinoma histology, five with embryonal carcinoma and four mixed. All of the testicle exams were normal but the pathology study found fibrous scarring in the majority of cases with haemotoxylin deposits in the seminiferous tubules, consistent with the burnt out phenomenon. In 1965, in a second publication on the subject, the same author presented a case that illustrated the* pattern of regression* in a testicular seminoma with viable metastasis. The author provides a comprehensive description of typical histological findings, as well as a comparison with choriocarcinoma [33].

In 1970, Veragut *et al* [34] reported two cases of young patients with a diagnosis of retroperitoneal seminoma with no evidence of a primary. In their discussion, they describe the ‘necrobiosis phenomenon’ as a means to explain the spontaneous involution of testicular tumours, indicating that it occurs more frequently in choriocarcinoma and is rare in seminoma.

In 1990 and 2000, two works were published, titled ‘Shrinking Seminoma’ and ‘Shrinking Seminoma—Fact or Fiction?’, describing volume reduction in the testicle with seminoma, where the mechanism is fundamentally ischemia—necrosis secondary to intermittent testicular torsion. Other possible causes also described were chronic inflammation and hormonal disorder. In the aforementioned phenomenon, depending on the stage at which it is diagnosed, a testicle ‘shrunken’ in size, with or without a residual tumour may be found, which is why in a patient with a retroperitoneal, mediastinal or other mass that also presents testicular shrinking, a GCT should be suspected [35, 36].

Near the end of the 20th century, various publications report on probable EGCT, in which lesions were found at the testicular level, consistent with spontaneous regression, correlating to the primary [5–9]. According to different publications, 90% of EGCTs occur between ages 20 and 35 and represent less than 5% of total GCTs; most commonly found in the anterior mediastinum, followed by the retroperitoneum and rarely in the pineal gland, presacral region or in another organ [7, 17, 20, 37, 38]. In general, every extragonadal tumour with GCT histology is considered a metastasis of hidden gonadal GCT until proven otherwise, with some authors even questioning the existence of EGCT [7].

Although the mechanism behind primary tumour regression has not been determined, there are several hypotheses. The two main hypotheses are: those related to an *immunological response* mediated by cytotoxic T lymphocytes that recognise tumour antigens and destroy malignant neoplastic cells, with subsequent fibrosis replacement; and those related to an *ischemic response* in the neoplasia, secondary to the blood supply deficit due to high metabolic rates and/or intermittent testicular torsion (Shrinking Seminoma). Another hypothesis indicates that when seminomas become metastatic, the organism produces antibodies that attack the metastasis, as well as the primary testicular tumour, which shrinks and may even be destroyed, leaving only traces. This foundation refers to the immunological theory of regression [26, 39–43].

Clinical manifestations generally depend on the metastatic disease [44–52]; only a few non-metastatic cases were diagnosed with signs and local symptoms such as pain in the scrotal sack, testicular shrinking and infertility studies. [26, 36, 43, 53, 54]. In accordance with the histology review performed, the most frequent symptoms were lumbar, abdominal and abdominal mass pain, very similar to the findings in our series, in addition to reports of weight loss. During the clinical evaluation of our case series, the first two patients were initially listed as having the lymphoproliferative syndrome and the third as having pulmonary metastasis of unknown origin. In various revised reports, due to various symptoms, the diagnostic work was also directed to pathology different from that of GCT [10–12, 14, 21, 24, 44, 54–57].

Examination of the scrotal sacs through palpation is insufficient to exclude testicular tumours. The findings depend on the size of the tumour, its relation to the size of the testicle, the placement, consistency and/or associated pathologies, such as hydrocele, cysts or others [7, 26, 28, 56, 58]. In our casuistry, the physical exam found no testicular tumours, supplemented with ultrasound.

The sensitivity of the testicular ultrasound for diagnosing GCT is close to 100%, so all young patients with a retroperitoneal or mediastinal mass should undergo this test. The characteristics of testicular tumour regression are not specific, as there have been findings of hyperechogenic, hypoechogenic and mixed lesions, nodular and linear areas, signs of testicular atrophy and/or acoustic shadowing reflecting calcifications or fibrosis [3, 26, 59, 60]. If the ultrasound results are inconclusive, scrotal magnetic resonance imaging (sMRI) can be useful in better defining these findings that are not necessarily malignant (infarction, ischemia, trauma or infection) [43, 59, 61]. Patel and Patel [53] reported that, when using the sMRI, a finding that suggests neoplasia is the appearance of a rapid higher height peak at the lesion. In the collection of histological data, it was found that the ultrasound findings most frequently related to regression were hypo- and hyperechogenic lesions and microlithiasis. Another useful imaging test for metastatic lesion detection that is also essentially in the control and follow-up after chemotherapy is positron emission tomography, along with multislice tomography (PET/CT) through radiopharmaceutical administration of F18-FDG [24, 28, 37, 62].

Tumour markers are fundamental to the diagnostic approach, staging, treatment and follow-up; they show up in varying forms depending on the histological lineage and the response to treatment. In our report, during the initial diagnostic, LDH was increased in all of the cases in relation to the metastasis and tumour burden, in two patients with HCG and in one with AFP [4, 5, 25, 38, 60].

The five reported cases were metastatic and diagnosed using the cytohistological results of the lesions, which, in addition to the clinical findings, laboratory and imaging studies, allowed us to formulate the primary testicular diagnosis and to indicate the corresponding radical orchiectomy. In the various reports, the macroscopic description of the testicles showing the partial or total tumour regression phenomenon, the presence of lesions that are hardened, whitened, fibrose, of scar aspect, in the form of nodules (singular or multiple), banded, linear or starred, is reported [3, 4, 7, 38, 63–65]. We found fibrosis scarring in every one of our cases.

Microscopically, our findings coincide with the WHO description defining the diagnostic criteria for testicular tumour regression, including inflammatory lymphoplasmacytic infiltrate (present in around 90% of cases), tubular hyalinization (around 70%), increase in vascularity (50%), hemosiderin (44%) and thick intratubular calcifications. The peripheral area was observed to have atrophy and sclerosis of the seminiferous tubules (100%), germinal cell malignancy *in situ* (approximately 50%), hyperplasia in Leydig cells (45%), intratubular microlitres (30%). The literature mentions intertubular calcifications and germinal neoplasia *in situ* as pathognomonic signs [3, 4, 38].

It is known that all germinal tumours have the potential for regression; however, there is evidence in the literature that disagrees on the frequency of the histological subtypes that demonstrate this phenomenon. Historically, choriocarcinoma has been considered the most prone to regression, but recent reports, as well as ours, confirm that seminoma has the most common histology, with exception of the spermatocytic type, which is now a separate entity. The teratomas are classified as the histology group with the lowest probability of regression [3, 4, 8, 38, 66, 67].

Various publications agree that chemotherapy is not completely effective for the testicles due to the haemato-testicular barrier and thus the necessity of surgical resection (orchiectomy) on the ‘regressed’ primary is essential and represents the cornerstone of the burned out definition, in addition to being the foundation for proper treatment. The management is, in general, similar to that for primary GCT testicles [6, 7, 56, 58, 59, 68–71].

For a large majority of patients, the diagnostic work was performed based on the symptomatology of the metastasis, not infrequently with an approach different to that of burned out GCT that shows the delay in proper diagnosis, the risk of complications due to time and progression of the disease, as well as procedures that some may have undergone. The incorrect extragonadal GCT diagnosis implies not treating the primary testicle, which may present partial regression, and, thus, in not responding to the systemic treatment and being maintained in a safe haven by the haemato-testicular barrier, it may become one of the most important factors determining recurrence and prognosis.

In our review of the literature, we found no conclusive publications on survival, the persistence of the disease or recurrence when compared to burned out GCT versus Gonadal and/or extragonadal GCT. In our case, the reported patients began a long-term follow-up protocol in order to carry out collaborative projects with other institutions in an attempt to answer the questions raised.

## Conclusions

Throughout time, the evidence regarding the ‘phenomenon’ of testicular tumour regression has been described in various publications, which has allowed for its current definition as an entity with its own diagnostic criteria.

Etiopathogenesis is still not well defined, nor if the tumoural regression tumour itself has some value in the prognosis. What is well defined is the indication for treatment in accordance with GCT protocols.

‘Burned out’ GCTs are classified as metastatic or non-metastatic with complete or partial regression. The most frequent ones were metastatic with complete regression and the most common histological type was seminoma.

In all tumours with GCT clinical and/or histology, the primary testicular tumour should be ruled out before classifying it as an extragonadal GCT.

## Conflicts of interest

The authors declared no conflicts of interest.

## Authors’ contributions

The main idea and literature review were by JCA and MAA; the collection of data was done by FMA, BAL and JLD. Manuscript revision and approval of the final copy was done by all.

## Figures and Tables

**Table 1.** Epidemiological and clinical data.

Case	Age (years)	Pathological history	Disease time	Principal signs and symptoms	Testicular examination	Admission Diagnosis
**1.**	54	No	6 months	Abd. tumour, lumbar and abdominal pain, WL.	Negative	Lymphoma
**2.**	58	No	2 months	Sc. and abd. tumour, lumbar pain, inc. vol. LEs, WL.	Negative	Lymphoma
**3.**	23	No	1 month	Haemoptysis and left WL hemiplegia	Negative	EAD Pulmonary Mets.
**4.**	36	No	7 months	Abd. tumour, lumbar pain, WL., incr. left LE vol.	Negative	Metastatic GCT
**5.**	20	No	3 months	Abd. tumour, abd. pain	Negative	Metastatic GCT

WL. Weight loss; Abd.: abdominal; SC: supraclavicular; LEs Incr. Vol.: enlargement of limbs; Mets: metastasis; EAD: aetiology to be determined.

**Table 2:** Imaging and tumour marker data at the initial diagnosis.

	Testicular ultrasound	CT abdomen/pelvis	CT thorax/brain	Doppler ultrasound vessels	Tumour markers		
					AFP (UI)	HCG (UI)	LDH (UI)
**1.**	RT with 23 mm× 26 mm × 12 mm. hypoec. nodule.	17 cm × 10 cm × 8 cm. RTP Tumour, encompasses aorta and collapses cava	No metastasis	No DVT	Normal	Normal	2480
**2.**	RT with 8 mm × 7 mm hypoec. nodule.	15 cm × 13 cm × 9 cm. RTP Tumour, encompasses the large vessels	Supraclavicular and mediastinal adenopathies	Cava and iliac DVT.	Normal	Normal	3637
**3.**	LT with 9 mm × 8 mm hypoec. pseudonodule	11 cm × 7 cm × 4 cm. RTP Tumour, encompasses aorta and iliac	Multiple pulmonary and frontoparietal Mets.	No DVT	Normal	19209	561
**4.**	LT with 30 mm. hypoec. node.	16 cm × 9 cm × 9cm RTP Tumour, encompasses large vessels	No metastasis	Iliac and left femoral DVT.	Normal	Normal	2480
**5.**	LT with 5 mm × 10 mm isoec nodule.	16 cm × 15 cm × 9 cm. RTP tumour in the left iliac region, encompasses vessels	No metastasis	No DVT	1745	3705	2948

RT: right testicle; LT: left testicle; hypoec.: hypoechogenic; RTP: retroperitoneal; DVT: deep vein thrombosis; Mets.: metastasis.

**Table 3.** Data on the management, anatomopathological diagnosis and state of the disease.

	Initial surgical management	AP (1)	Second procedure	AP(2)	Adjuvant Therapy	Time of follow-up	Status disease
**1.**	RTP tumour biopsy	Seminoma	Radical Orchiectomy	Fibrous scar	Chemo (BEP x 4)	53 months	NED
**2.**	Supraclavicular tumour biopsy	Seminoma	Radical Orchiectomy	Fibrous scar	Chemo (BEP x 4)	40 months	NED
**3.**	Pulmonary nodule biopsy	Choriocarcinoma	Radical Orchiectomy	Fibrous scar	Chemo + WBRT	7 months	DOD
**4.**	Radical Orchiectomy	Fibrous scar	RTP tumour biopsy	Seminoma	Chemo (BEP x 4)	16 months	NED
**5.**	Radical Orchiectomy	Fibrous scar	RTP tumour biopsy	Mixed (EC/YST/T)	Chemo (BEP x 4)	3 months	AWD

EC: embryonal carcinoma; YST: yolk sac tumour; T: teratoma; WBRT: whole brain radiotherapy; BEP: bleomycin/etoposide/platinum; NED: no evidence of disease; DOD: dead of disease; AWD: alive with disease; AP: anatomical pathology.

**Table 4.** Anatomopathological results of the metastasis, diagnostic procedure and correlation with tumour markers.

	Anatomopathological diagnosis	Diagnostic procedures	Altered tumour markers
**1.**	GCT (Seminoma)	IOC-SBx of RTP tumour	LDH
**2.**	GCT (Seminoma)	FNAB-SBx of supraclavicular tumour	LDH
**3.**	GCT (Choriocarcinoma)	FNAB of lung tumour	HCG; LDH
**4.**	GCT (Seminoma)	RTP tumour biopsy	LDH
**5.**	Mixed GCT (EC/YST/T)	IOC-SBx of RTP tumour	HCG; AFP; LDH

IOC: Intraoperative cytology; SBx: Surgical biopsy; RTP: retroperitoneum; FNAB: Fine needle aspiration biopsy; EC: embryonal carcinoma; YST: yolk sac tumour; T: teratoma.

**Table 5.** General data from the 2000–2018 literature review.

1. Total Publications	57	
2. Total Cases	159	
3. Average age/range (years)	35.96	17 - 67
4. Pathological history:		
- Cryptorchidism	9	(15.8%)
- Contralateral GCT	2	(3.5%)
5. GCT burned out:		
- Metastatic	154	(96.8%)
- Non-metastatic	5	(3.2%)
6. Testicular tumour regression:		
- Complete	114	(71.7%)
- Partial	45	(28.3%)
7. Affected testicle:		
- Right	74	(47%)
- Left	67	(42%)
- Undetermined	18	(11%)
8. Histological type of GCT:	In metastasis	In the testicle
- Pure seminoma	81 (50.8%)	23 (53.5%)
- Mixed with seminoma	12 (7.4%)	8 (18.7%)
- Mixed without seminoma	17 (11.1%)	3 (6.9%)
- Pure embryonal carcinoma	16 (10.1%)	2 (4.6%)
- Pure Choriocarcinoma	4 (2.5%)	0
- Pure yolk sac tumour	4 (2.5%)	1 (2.3%)
-Teratoma	5 (3.1%)	6 (14%)
- Undetermined	20 (12.5%)	NA
- Total	159 (100%)	43 (100%)

**Table 6.** Publications on spontaneous testicular GCT regression (2000–2018)

No.	Author (Year)/bibliographic Ref. No.	No. cases	Age/Average (years)	GCT		6. Testicular tumour regression:	
				Metastatic	No metastatic	Complete	Partial
1	Leleu *et al* (2000) [44]	1	34	1		1	
2	Naseem *et al* (2000) [36]	2	34	1	1	1	1
3	Scholz *et al* (2001) [7]	26	36	26		22	4
4	Kebapci *et al* (2002) [72]	1	22	1		1	
5	Bissen *et al* (2003) [39]	1	33	1		1	
6	Tasu *et al* (2003) [59]	5	31	5		3	2
7	Fabre *et al* (2004) [26]	5	34.6	4	1	4	1
8	Mola *et al* (2005) [69]	1	33	1		1	
9	Perimenis *et al* (2005) [45]	1	40	1		1	
10	Castillo *et al* (2005) [68]	1	25	1		1	
11	Curigliano *et al* (2006) [46]	1	42	1		1	
12	Balzer and Ulbright (2006) [3]	42	32	42		26	16
13	Yamamoto *et al* (2007) [10]	1	39	1		1	
14	Parada *et al* (2007) [64]	2	19.5	2			2
15	Patel and Patel (2007) [53]	1	23		1		1
16	Vasquez *et al* (2008) [56]	3	38	3		3	
17	Coulier *et al* (2008) [11]	1	53	1		1	
18	Angulo *et al* (2009) [38]	17	31	17		10	7
19	Kontos *et al* (2009) [40]	1	31	1		1	
20	Ha *et al* (2009) [47]	1	23	1		1	
21	Yucel *et al* (2009) [48]	1	28	1		1	
22	Yucel *et al* (2009) [49]	1	49	1		1	
23	Mesa *et al* (2009) [12]	1	55	1		1	
24	Orlich and Jimenez (2010) [50]	1	33	1		1	
25	Gaytán *et al* (2010) [51]	1	19	1		1	
26	Jaber S. (2010) [52]	1	32	1		1	
27	Womeldorph *et al* (2010) [73]	1	55	1		1	
28	Musser *et al* (2010) [13]	1	63	1			1
29	Herrera *et al* (2011) [14]	4	33	4		3	1
30	Balalaa *et al* (2011) [57]	1	31	1		1	
31	Kar *et al* (2011) [15]	1	33	1		1	
32	Preda *et al* (2011) [70]	1	43	1			1
33	Gonzales *et al* (2012) [16]	1	35	1		1	
34	Peroux *et al* (2013) [60]	1	18	1		1	
35	Gurioli *et al* (2013) [58]	2	42.5	2		2	
36	Sahoo *et al* (2013) [41]	1	33	1		1	
37	Ichiyanagi et al (2013) [43]	1	47		1	1	
38	Miacola *et al* (2014) [42]	1	36	1		1	
39	Chung *et al* (2014) [54]	1	33		1		1
40	Onishi *et al* (2013) [18]	1	41	1		1	
41	Qureshi *et al* (2014) [71]	1	20	1			1
42	Budak *et al* (2015) [55]	1	39	1		1	
43	McCarthy *et al* (2015) [63]	1	24	1		1	
44	Gomis *et al* (2015) [74]	1	42	1		1	
45	Nguyen *et al* (2015) [62]	1	64	1			1
46	Hu *et al* (2015) [75]	1	37	1		1	
47	George *et al* (2015) [19]	1	24	1			1
48	Ishikawa *et al* (2016) [21]	1	42	1		1	
49	El sanharawi *et al* (2016) [61]	5	37	5		5	
50	Iwatsuki *et al* (2016) [76]	1	29	1			1
51	Nakazaki. *et al* (2016) [65]	1	54	1			1
52	El-sharkawy and Al-Jibali (2017) [78]	1	22	1		1	
53	Juul and Rasmussen (2017) [22]	1	57	1			1
54	Mosillo *et al* (2017) [28]	1	19	1			1
55	Nishisho *et al* (2017) [23]	1	30	1		1	
56	Ulloa-Ortiz *et al* (2017) [77]	1	52	1		1	
57	Freifeld *et al* (2018) [24]	1	44	1		1	
		159	35.9	154	5	114	45
